# Buccally displaced flap versus sub-epithelial connective tissue graft for peri-implant soft tissue augmentation: a pilot double-blind randomized controlled trial

**DOI:** 10.1186/s40729-020-00244-4

**Published:** 2020-09-01

**Authors:** Ninad Milind Padhye, Lopa Kishor Mehta, Naveeta Yadav

**Affiliations:** 1 Ceramco Dental Care, Andheri West, Mumbai, Maharashtra India; 2 King Edward Memorial Hospital, Mumbai, Maharashtra India; 3 Mahatma Gandhi Postgraduate Institute of Dental Sciences, Pondicherry, India

**Keywords:** Connective tissue graft, Displaced flap, Peri-implant keratinized mucosa, Soft tissue augmentation, Surgical flaps

## Abstract

**Background:**

This article describes a novel surgical technique, the buccally displaced flap, for keratinized mucosa (KM) augmentation during implant uncovering. Furthermore, it clinically compares this technique with sub-epithelial connective tissue graft (SCTG) for peri-implant KM augmentation. Twelve weeks following implant placement, subjects were randomly divided for KM augmentation into group A (buccally displaced flap) and group B (SCTG). The width (WKM) and thickness (TKM) of the KM were assessed prior to the implant uncovering, 4 weeks and 1 year after implant loading. Post-operative pain assessment was performed using the Numeric Rating Scale.

**Results:**

The study comprised of 20 implants that were uncovered in 20 subjects. For group A, the mean WKM increased from 0.98 (± 0.23 mm) to 3.01 mm (± 0.18 mm), and the mean TKM increased from 1.45 (± 0.13 mm) to 2.21 mm (± 0.16 mm) at 1 year. For group B, the mean WKM increased from 0.93 (± 0.18 mm) to 3.28 mm (± 0.13 mm), and the mean TKM increased from 1.41 (± 0.15 mm) to 2.25 mm (± 0.11 mm) at 1 year. Post-operative pain was significantly higher for group B 4.15 (± 1.35) as compared to group A 2.6 (± 1.22) (*p* < 0.001).

**Conclusion:**

The buccally displaced flap increased the WKM and TKM during implant uncovering, with results comparable to SCTG. The main advantages of the technique were lack of sutures, maintenance of blood supply, reducing number of surgical sites, and it was relatively atraumatic with lesser post-operative pain.

**Trial registration:**

Clinical trials registry—India CTRI/2019/09/021059. Date of registration—September 4, 2019, retrospectively registered.

## Background

Hard and soft tissue deficiencies are a common occurrence as a result of long-term edentulism. Following tooth extraction, the degree of bone resorption can reach up to 50% of the original bone width in the first 2 years [1–3]. With substantial bone loss, a coronal shift in the mucogingival junction is also noted [4]. This potentially compromises the keratinized tissue dimensions for implant-based rehabilitations.

Although the role of keratinized mucosa (KM) for implant survival is still debatable, it has been established that the absence of KM impedes adequate oral hygiene maintenance [5]. An indirect correlation has been found between KM dimensions and plaque accumulation and gingival inflammation [6].

Traditionally, sub-epithelial connective tissue graft (SCTG) was harvested from the hard palate for peri-implant soft tissue augmentations. However, this procedure often required a secondary surgical site to procure the autogenous graft, adding to the post-operative discomfort. Abrams [7] introduced the roll flap technique for soft tissue augmentation for edentulous ridges. This technique provided a vascular connective tissue pedicle graft that could minimize the donor site morbidity. Subsequently, several techniques have been suggested for implant site soft tissue augmentation such as modified palatal roll flap [8], apically positioned flap [9], split-finger flap [10], tunnel exposure [11], and rotated palatal flap [12]. This study aims to describe the buccally displaced flap, a novel surgical technique for implant site soft tissue augmentation. Furthermore, it compares this technique to SCTG for peri-implant KM augmentation during implant uncovering.

## Materials and method

This prospective double-blinded randomized controlled clinical trial was conducted in accordance with the ethical standards outline in the 1964 Declaration of Helsinki, as revised in 2008. Ethical clearance was attained, and the study was registered with the clinical trials registry.

### Surgical technique for buccally displaced flap (supplementary video)

**Fig. 1:**
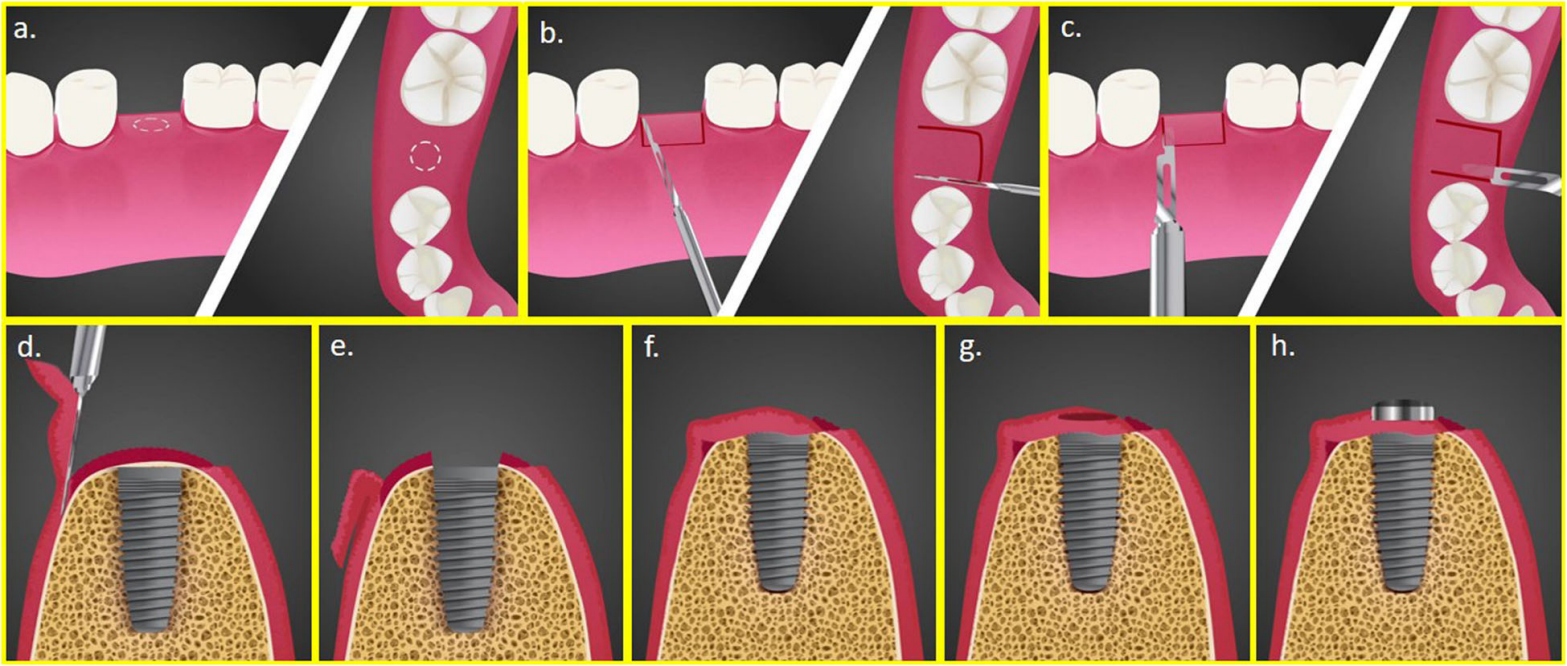
Buccally displaced flap surgical technique. **a** Bone sounding. **b** Two parallel and one perpendicular incisions placed. **c** Partial thickness tissue dissection underneath the incisions. **d** Tissue dissection extending till the mucogingival junction on buccal side, and partial thickness flap reflected. **e** Connective tissue over the implant removed. **f** Partial thickness flap displaced buccally and adapted over the surface. **g** 3 mm tissue punch. **h** Healing abutment to secure the flap in its new position

The surgical procedure was carried out during the implant uncovering stage 12 weeks after implant placement. Under local anaesthesia, bone sounding was performed on the crest of the ridge (Fig. 1a) using a UNC-15 probe (Hu-Friedy, USA), and the soft tissue thickness over the previously placed implant was found to be within the range of 2–4 mm. Two parallel vertical incisions were placed, extending 3–4 mm on the palatal/lingual surface of the edentulous crest. The parallel incisions were connected by a perpendicular incision on the palatal/lingual side (Fig. 1b). Partial thickness tissue dissection was then performed underneath the incisions extending till the mucogingival junction on the buccal side, and a partial thickness flap was reflected (Fig. 1c, d). The connective tissue over the implant was removed, and the implant was exposed using a curette (Hu-Friedy, USA) (Fig. 1e). The partial thickness flap was then displaced buccally and adapted over the surface (Fig. 1f). A 3-mm tissue punch was made in the displaced flap over the implant surface (Fig. 1g). To secure the flap in its new position, a 3–5-mm high healing abutment was placed through the punched flap into the implant (Fig. 1h).

### Surgical technique for sub-epithelial connective tissue graft

Twelve weeks after implant placement, under local anaesthesia, a crestal incision was placed at the implant site. A full thickness mucoperiosteal flap was reflected to expose the implant. A 3–5-mm high healing abutment was placed into the implant. SCTG was harvested from the palate with a single incision technique and tucked buccally underneath the reflected flap. The graft and the flap were then stabilized using interrupted sutures.

### Study design

Subjects were recruited for the study with the inclusion criteria: (1) > 21 years of age, (2) no medical contraindications and generally healthy patients, (3) missing teeth in the posterior region (premolar/molar), (4) no bone augmentation procedures required before or during implant placement, and (5) signed informed consent form for participation and permission to use obtained data for research purposes. Patients were excluded if they had (1) poor oral hygiene, (2) smoking or tobacco chewing habit, and (3) diabetes mellitus.

**Fig. 2:**
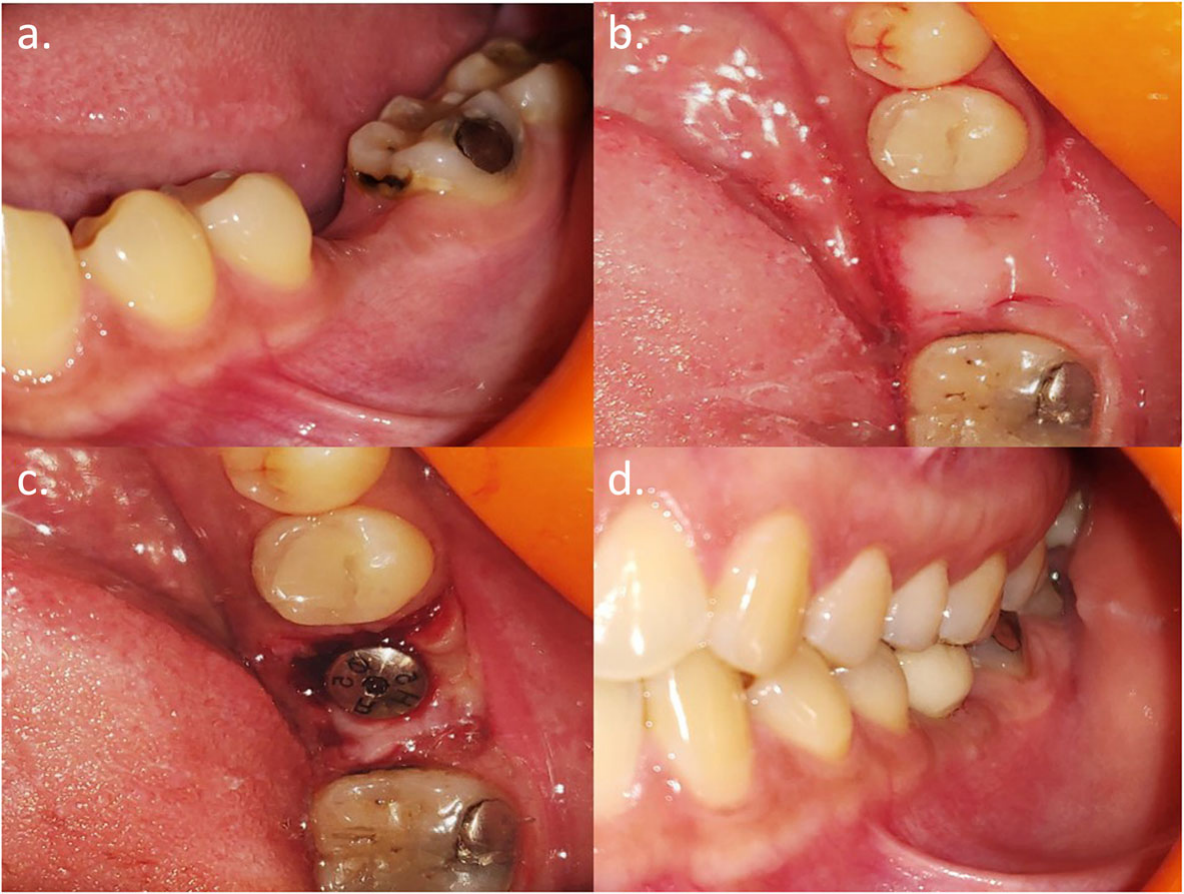
Peri-implant keratinized mucosa augmentation for group A. **a** Pre-operative. **b** Incisions placed. **c** Flap displaced buccally and stabilized by a healing abutment. **d** 1 year follow-up

**Fig. 3:**
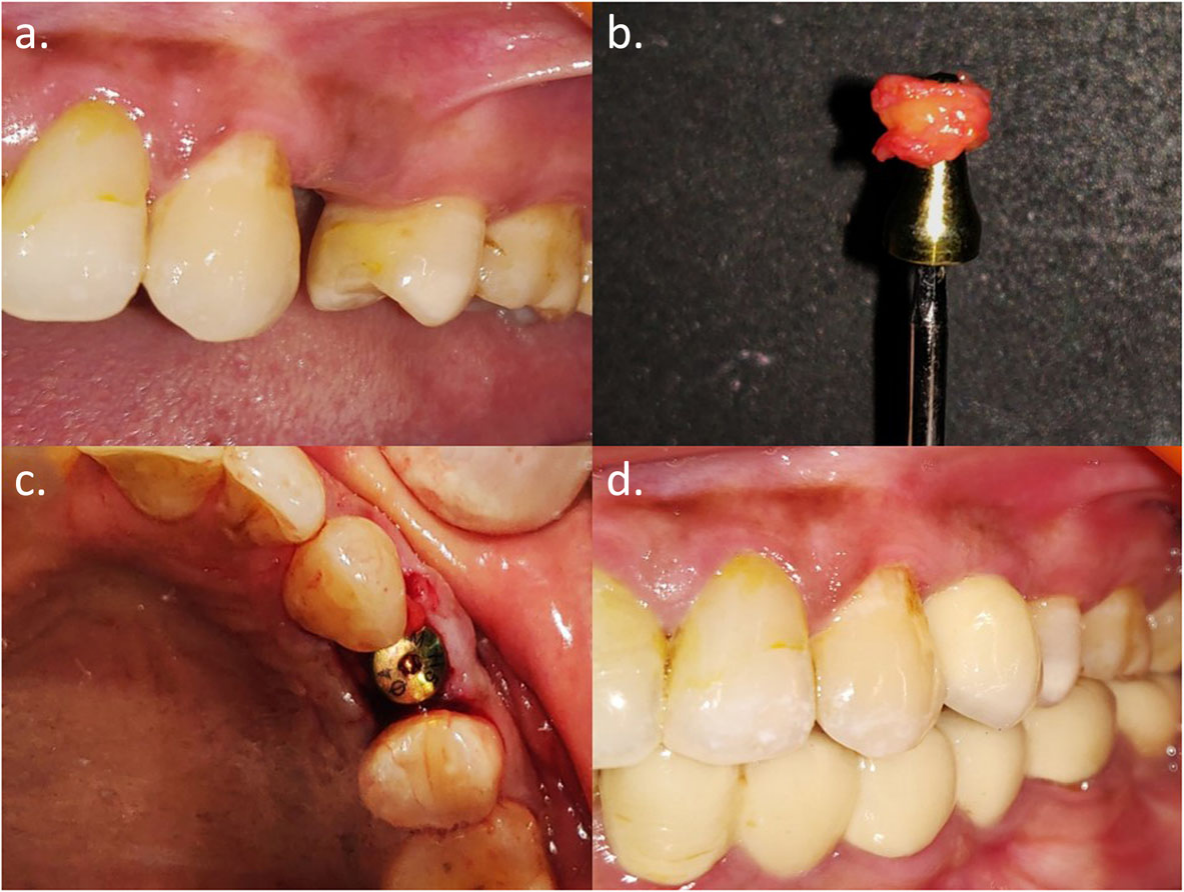
Peri-implant keratinized mucosa augmentation for group B. **a** Pre-operative. **b** Sub-epithelial connective tissue graft harvested from hard palate. **c** Graft tucked buccally and healing abutment placed. **d** 1 year follow-up

A standard sample size calculation determined that a minimum of 10 subjects per group with a total of 20 subjects were required for the difference between WKM and TKM values to be statistically significant when *α* = 0.05, the power of the study set at 80%, as determined by a study done by Temmerman et al. in 2018 [13]. Two-stage bone-level endosseous implants (Osstem TSIII, Osstem Implant Co., Busan, Korea) were placed in 20 subjects included for the study. Twelve weeks after the implant placement surgery, subjects were randomly divided into two groups, by envelope drawing, for soft tissue augmentation. Subjects of group A received augmentation using the buccally displaced flap (Fig. 2), and subjects of group B received augmentation using SCTG (Fig. 3) respectively. Treatment assignment was noted in the registration by the study monitor (N.Y.). Allocation concealment was performed using sequentially numbered sealed envelopes that were made by the study monitor using computer-generated random permuted block. The randomization envelope was opened prior to the surgical procedure. All the surgical procedures were carried out by a single operator (N.P.). Post-operatively, 400 mg ibuprofen was prescribed to all the participants on an as-needed basis.

The width (WKM) and thickness (TKM) of the KM were the clinical parameters assessed for the included subjects. WKM was measured on the buccal aspect from the gingival margin to the mucogingival junction using a UNC-15 probe (Hu-Friedy, USA). The TKM was measured 2 mm apical to the free gingival margin on the buccal aspect using an endodontic reamer file. The parameters were assessed prior to the implant uncovering (baseline), 4 weeks and 1 year after implant loading. Post-operative pain assessment was performed 1 week following the implant uncovering using the Numeric Rating Scale [14, 15]. The participants were instructed to select a number from 0 through 10 that best reflected the intensity of pain, with 0 equalling no pain and 10 the worst pain. All the clinical assessments were done by a second operator (L.M.) who was blinded to the surgical procedure performed.

### Statistical analysis

The recorded data was entered in Microsoft Excel (MS office version 2010) and tabulated. Data analysis was done using the Windows PC based software “MedCalc Statistical Software” version 13.3.1 (MedCalc Software bvba, Ostend, Belgium; http://www.medcalc.org; 2014). All testing was done at alpha 0.05 (95% confidence limits). Intra- and inter-group comparison was performed using the paired and unpaired student *t* test, respectively, considering normality assumption and homoscedasticity. Differences above the 95% confidence intervals were regarded as statistically significant.

## Results

For this pilot study, a total of 20 implants placed in 20 subjects (13 females and 7 males) were included. The mean age of the study participants was 48 ± 5.9 years. No implants were lost or showed signs of peri-implantitis during the follow-up period of 1 year.

**Fig. 4:**
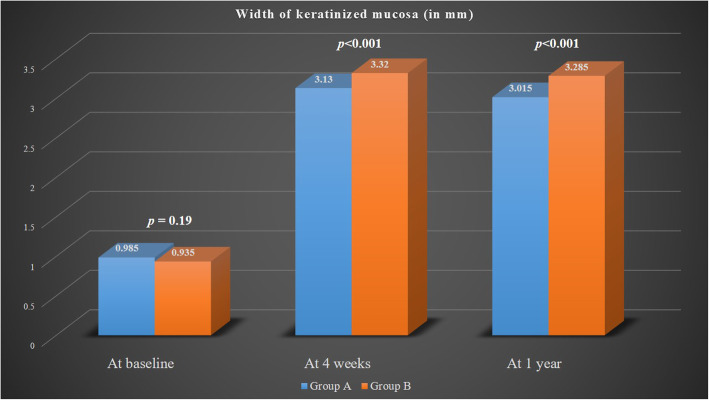
Comparison of the width of keratinized mucosa between group A and group B at various time points

At the time of implant uncovering, there was minimal WKM noted (group A, 0.98 mm ± 0.23 mm; group B, 0.93 mm ± 0.18 mm) (*p* = 0.19). At 4 weeks and 1 year after implant loading, the WKM significantly increased for both the groups from baseline. The WKM at 4 weeks and 1 year for group B (3.32 mm ± 0.19 mm, 3.28 mm ± 0.13 mm) was significantly higher than group A (3.13 mm ± 0.22 mm, 3.01 mm ± 0.18 mm) (*p* < 0.001) (Fig. 4).

**Fig. 5:**
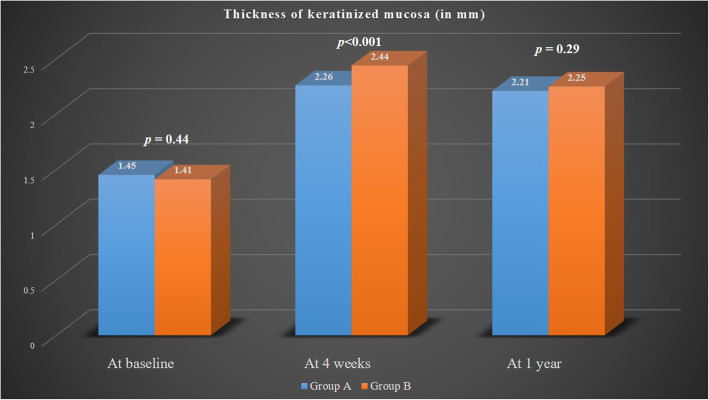
Comparison of the thickness of keratinized mucosa between group A and group B at various time points

The mean TKM at baseline was 1.45 mm ± 0.13 mm for group A and 1.41 mm ± 0.15 mm for group B (*p* = 0.44). At 4 weeks, the TKM was considerably higher for group B (2.44 mm ± 0.26 mm) as compared to group A (2.26 mm ± 0.31 mm) (*p* < 0.001). However, at 1 year, there was no statistically significant difference in TKM between group B (2.25 mm ± 0.11 mm) and group A (2.21 mm ± 0.16 mm) (*p* = 0.29) (Fig. 5). Post-operative pain and discomfort according to the Numeric Rating Scale were significantly greater for subjects of group B (4.15 ± 1.35) as compared to group A (2.6 ± 1.22) (*p* < 0.001).

## Discussion

Peri-implant soft tissue augmentation can be performed at various stages during the dental implant procedure such as prior to implant placement, in combination with bone augmentation, simultaneous with implant placement, at the temporization phase, during the phases of osseointegration, prior to delivery of the final reconstruction, or during the second stage surgery [16]. The proposed surgical procedure aimed at correcting the buccal keratinized mucosa deficiencies during the implant uncovering phase. Maintaining an adequate thickness of the marginal gingiva at an early phase of the implant uncovery is essential for the maintenance of peri-implant health and esthetics [17]. Transformation of a thin gingival biotype to thick gingival biotype results in a stable peri-implant soft tissue dimension [18]. An adequate width of keratinized mucosa attached to the underlying periosteum is essential for the overall long-term success of implant supported rehabilitation [12, 19].

The buccally displaced flap surgical technique was a combination of the Abram’s roll technique [7] and the apically displaced flap [20]. It employed the use of a partial-thickness flap, leaving behind 0.5–0.75 mm of connective tissue and periosteum layer over the surrounding bone. This prevented the alveolar crestal bone loss and recession that usually occur following the reflection of a full-thickness mucoperiosteal flap [21]. Additionally, dual blood supply to the flap was ensured, from the underlying periosteal vasculature as well as from the flap’s supraperiosteal vessels [22]. An epithelium-denuded surgical wound of 2–3 mm (depending on the extent of tissue displacement) was created on the palatal/lingual side of the surgical bed that healed by secondary intention.

A healing abutment firmly secured the flap, through a punch hole, and provided primary stability during its healing process. The final thickness of the keratinized mucosa depended on the thickness of the displaced flap. A follow-up period of 1 year was considered for this study, as maximum soft tissue changes occur around a dental implant during the first 6 months to 1 year [23]. Mild to moderate localized buccal horizontal depression (Seibert Class 1 ridge deficiency) [24] was corrected using this augmentation technique. Regardless of the soft tissue augmentation technique used, some degree of relapse and shrinkage of the initially augmented keratinized mucosa is usually observed [25], as was seen in the subjects of our study at the 1 year follow-up visit.

A significant difference was seen in the post-operative pain, where the buccally displaced flap group experienced lesser discomfort than the sub-epithelial connective tissue graft group. This may be attributed to the fact that the buccally displaced flap technique did not need a secondary donor surgical site and was relatively atraumatic. Post-operative healing was devoid of the risk of slough formation of the superficial split-palatal flap, and thus, palatal pain. Furthermore, surgical procedures without sutures are usually considered minimally invasive by the patients and have a better acceptance [26].

In the present study, all procedures were performed by one surgeon. Thus, a surgeon-related bias may have been introduced as the surgeon has some “preferred” treatment procedure [27]. The limitations of this technique may include operator skill and experience in mucogingival surgical procedures. A high risk of flap perforation is also present in patients with a thin gingival biotype. Furthermore, although the study design was double-blinded, the subjects in group B had an additional surgical site (the hard palate) from where the sub-epithelial connective tissue graft was harvested, and thus were aware of the group they belonged to.

## Conclusion

Within the limitations of the study, it was seen that the buccally displaced flap technique increased the width and thickness of the keratinized mucosa around implants over the observation period of 1 year, which was comparable to the subepithelial connective tissue graft. The surgical technique caused minimal patient discomfort and may be routinely employed to augment the peri-implant soft tissues.

## Supplementary information

**Additional file 1.** Buccally displaced flap surgical technique

## Data Availability

Data sharing is not applicable to this article as no datasets were generated or analysed during the current study,
